# Tracts on Generation; The Medical Practioner and Student’s Library

**Published:** 1848-03

**Authors:** 


### BIBLIOGRAPHICAL NOTICES.

*Tracts on Generation.* Translated from the German. By C. R. Gilman, M. D., Professor of Obstetrics, &c., College of Physicians and Surgeons, New York; and Theodore Tellkampf, M. D., Gebhard Professor, Columbia College. Number 1. *Proofs that the Periodic Maturation and Discharge of Ova are, in the Mammalia and the Human Female, Independent of Coition, as a First Condition of their Propagation.* By T. L. G. Bischoff, Professor of Physiology, &c., Giessen. 8vo. pp. 65.

*The Medical Practitioner and Student's Library. The Principles and Practice of Midwifery.* By David H. Tucker, M. D., Professor of the Principles and Practice of Medicine, &c. &c., with numerous Illustrations. Small 8vo., pp. 405. Philadelphia, 1848.

We are, in certain respects, a singular people; some of us, that is. Whilst the nature of our institutions would—it might be presumed—irresistibly impel us to regard all men alike, or make us pause before we unduly elevated one man far above another, nowhere, perhaps, do we find more numerous examples of recklessness in this respect, and of the influence of *cliquism* or favouritism of some form, under which men are *bruited* abroad as "distinguished," "eminent," although perhaps comparatively unknown, except in their own locality. The same, or a similar feeling, leads to individuals being designated as the "unquestioned heads"—the "most distinguished members" of their profession. There is evil in all this. It is, to say the least, invidious: and the very epithet "unquestioned" must almost always—if not always—be translated to really mean "questioned." Most certainly we do not recollect to have seen it applied, except where a question might be entertained of its truth; and occasionally it has been directed—we have been so impressed at least—towards persons who ought themselves to doubt whether the epithet were employed in all seriousness—regard being had to the public evidences they have exhibited of their fitness for it. We believe that there is real evil—as we have said—in this glorification. We should prefer that the republic of science should have no single head;—that there should be many heads to it, each holding co-ordinate rank. We would know of no such thing as British or French, or German, or American science. The realm of science should admit of no sections or subdivisions. It should be one and indivisible; and whilst glory should wait on the efforts of those who have succeeded in extending its boundaries; it should be a glory awarded not by sectional but by cosmopolitan voices; otherwise an invidious question might arise—which ought never to be permitted—as to the comparative value, for example, of a reputation for Philadelphia, or New York, or Boston science; and to an attempt to elevate in every community "unquestioned heads" in medicine, natural science generally, physical and moral science, &c., which would tend to a perpetual strife and confusion; a paralyzing of energies; and the detriment of science. If in our ordinary parlance, we were to estimate and to speak of an individual as "*one* of the most distinguished men of science," "*one* of the unquestioned heads," we should be nearer the truth; and assuredly less invidious.

The same kind of feeling induces us to extol unmeasuredly, and as certainly to spoil, those who come among us from abroad, after having exerted themselves successfully for the furtherance of knowledge; and there are few of the actors in the theatrical laudations of one, who visited this country almost as a literary hero, some years ago, and who was received with shaking of hands, and *feted* in every city through which he journeyed, who are not heartily ashamed of the part they played on the occasion. Yet individuals may be benefited by experience; masses rarely are; and when a like occasion presents itself, we shall doubtless have the same scene acted over again.

These remarks are suggested by a letter, contained in the work whose title is first given at the head of this article. For the name and services of M. Agassiz—the distinguished naturalist—no one has a greater respect than ourselves. Yet we consider the extravagant eulogies that have been passed upon him since his arrival among us, to be—to say the least of them—wanting in good taste, and we doubt not that they have been as far from agreeable to the estimable and unassuming naturalist as they have been wanting in true dignity.

In a letter addressed by M. Agassiz to Dr. Gilman, he suggests " the propriety of translating into English, the pamphlet of Dr. Bischoff, upon Ovulation and Fecundation of the Mammalia," which he characterizes as a " model in this kind of experiments," and expresses a hope, that by introducing the work of his learned friend before our physicians, " it will become a link the more in the pleasant intercourse which already exists between the scientific men of the two countries, and start some of our young men in the line of original investigation in this important department."

M. Agassiz has been too short a time among us to know what has been published in this country, or to be aware, that with every observing, reflecting, and reading physician the name and labours of M. Bischoff are as familiar as their household gods; but the American editors of this " tract"—one of them especially—the Professor of Obstetrics, &c., in the College of Physicians and Surgeons, New York—ought to have been aware of the fact. " The letter of Agassiz"—say the Editors—" which accompanies this, will explain the reason why this translation was made, and commends it to the notice of American physiologists more powerfully than any word of ours could do:"—and they add, " This tract is issued as No. One of " Tracts on Generation " *translated from the German*," and we desire and design if our publishers are encouraged to go on with the work, to follow this by translations of other of the more interesting monographs on generation, which have appeared in Germany during the last few years. Ovology and Embryology, may without exaggeration, be called *German sciences*," [the Italics are in the original] " and we cannot doubt, that American physicians will be glad to possess, in their own language, the original researches of those who have taken the lead in these important subjects. In the series we propose, original researches only will have place, and when we say that in making our selection we shall have the aid of Agassiz, we give the strongest assurance that they will be judiciously made." And then comes a sentence, which shows, in addition to one or two matters, on which we shall have to animadvert, how strangely defective the Editors must be in their acquaintance with modern medical literature. " It is proper to state, that a translation of the greater part of this tract appeared in detached portions in the London Medical Gazette of 1845–6. *Of the existence of this translation we were not aware till ours was in progress, and we have not availed ourselves of it.*" p. vi.

But, in the first place we may ask, on what authority do the editors affirm that "ovology and embryology may, without exaggeration, be called *German sciences.*" That we are largely indebted to German observers on these subjects no one can question; but a question can as little arise as to our indebtedness to those of France and England. No one can refer to the pages of a cosmopolitan work—if we may be permitted to employ the epithet for one whose object it is to do justice to the labouring men of all nations—on physiology, without perpetually striking upon the names of distinguished individuals of other countries, whose investigations have effected as much for the advancement of ovology and embryology as those of Germany. We will not follow the vicious custom of adducing a string of names to support this assertion, under the fear that, by omission, we may unintentionally do injustice to some member of the community of science, eminently worthy of notice: but we say again, let the reader refer to any physiological treatise, written in our own country, by one who is himself acquainted with all that has been done elsewhere, and ready to do justice to all—and we believe that many of our works are more cosmopolitan in their character than those of other countries—and he will have some difficulty in deciding as to what nation—if the invidious question be raised—has done most to make the science of ovology and embryology its own. The German, the French, the English, may have a bias to their own country. Young and ardent neophytes—not perhaps eminently capable of judging when they entered—and still less so when they left—the glare of the capitals of Europe—

> "Returning from their finished tour,
> Grown ten times perter than before,"

may give their decision for the one or the other, according to impressions received when abroad; but the sober and sound-thinking individual, will be compelled to award praise to all, and to avoid offensive comparisons. Even on this very subject of ovulation, or of "the maturation and discharge of ova, independent [independently] of coition"—and who, at the present day, requires any proof of this fact ?—M. Bischoff refers to the labours of Raciborski, Duvernoy, Pouchet, Négrier, and Gendrin, all *Frenchmen*, and to Messrs. Wharton Jones, Dr. Lee, and Dr. Paterson,—*Englishmen*, whilst he does not notice the observations of a single German, on the subject of what the editors designate "German science." Nay, at this very time, and both in the recently published and most extensive work on the subject of ovulation that has appeared, by M. Pouchet, and in the very "tract" before us, a controversy is maintained as to the precise individual, Bischoff, Pouchet, or Raciborski, who first established the connection between ovulation and menstruation,—a subject, by the way, still requiring investigation, and very far, in our opinion, from being settled in accordance with the views of those who are the most dogmatic on the subject. We would, indeed, suggest to the editors of this "Tract" and Series of Tracts, should the publishers be encouraged to go on with the work, which we seriously apprehend they will not, to re-publish the valuable articles of Dr. Ritchie, of Glasgow, which—as they do not appear to see the *London Medical Gazette*—we would inform them, are contained in that journal for 1844, in order that they, who read M. Bischoff's views on the maturation and discharge of ova, as connected with menstruation, and menstruation only, may learn, that ample evidence of the most forcible kind is afforded by him—the result of attentive and multiplied observation—that even during childhood, and in the intermenstrual periods, unfecundated but maturated ovules leave the ovary.

We cannot, however, assign much more space to this notice. We may add, that in the work of Dr. Tucker—written before the publication in this country of the "Tract" before us—we find much space given to the experiments and observations of M. Bischoff among others; to the vexed question of the connection between ovulation and menstruation; the asserted "impossibility"—according to M. Pouchet—a most hazardous and unproved position—of impregnation ensuing after the first twelve [or fourteen] days from the cessation of the catamenia—the ovule, according to that gentleman, requiring twelve days, and rarely fourteen, for it to be discharged with the decidua. We have said, that the doctrine of M. Pouchet is a hazardous one. It is really less so, however, as shown by Dr. Tucker, in a note to his chapter on generation, than it might appear to be at first sight.

"The case," says Dr. Tucker, "extracted from the work of Dr. Dewees, whose authority cannot be questioned, proves that a female may be impregnated one week before the occurrence of the catamenia: while Pouchet, &c. assert that impregnation may occur as late as twelve days" [and even fourteen days] "after the menstrual flow. Now if these twelve days of susceptibility be added to the number of days previous to menstruation, during which fecundation may occur, as ascertained by Dr. Dewees, we will have nineteen days out of the twenty-eight, during which a female may be impregnated. But more than this, if we add to these nineteen days four or five days for the duration of the menstrual flow, it will give us twenty-three or four days out of the twenty-eight, during which a female is capable of impregnation, leaving only four or five days" [two or three] "as the length of time during which it is impossible. It may be objected, that Dr. Dewees asserts, that the female was "within a week of her menstrual period," and therefore we have no right to assume that it was seven days previous to the catamenia. To this we would reply, that the case *certainly* proves that impregnation may occur before the catamenia, and is, therefore, in any point of view, destructive of the assertion laid down by Pouchet. But this is not our only proof. M. Raciborski states, that fecundation may occur a few days before the catamenial flow; and Montgomery has reported a case in which it was accomplished three days before the catamenia commenced." p. 74.

As Dr. Tucker's work, by the way, is open before us at the very place, we may cite his summary of the theory of generation —a most intricate subject on which obscurity rests—not dispelled, we think, by his statement, which is itself far from being classically expressed, or characterized by what we would deem to be sound physiology.

"The theory of generation may," he says, "be summed up as follows: the female furnishes the germ, egg or ovule: which pre-exists, or is produced, in indefinite numbers in the ovarium; which maturates at certain periods, ruptures its natural covering, and is brought into contact with the seminal fluid, either at the ovary, or in the oviduct, or in the uterus. The male, on the contrary, furnishes a fluid, composed not of anamalcules" [M. Pouchet, in his late work, has endeavoured to exhibit that they are animalcules, and has given drawings to show their organization] "but of bodies having the power of motion, like the epithelial cells, &c.; that these bodies are carried by an unknown power to the Fallopian tubes or even to the ovaries; when meeting with the ovule divested of its coverings, by a union of the two, how, we know not, *vitality results*" [?]; the *being* thus formed being composed of principles from each parent, will at times present traits of resemblance, both moral and physical, to either one or the other, or perhaps both parents." p. 75.

Of Dr. Tucker's "*Elements of the Principles and Practice of Midwifery*"—[we do not like the term " Elements of the Principles"] we are happy in being able to speak in language of approbation. The limits assigned to him obviously precluded a full consideration of every subject; and, being his first production, it was not—could not be—easy for him to be entirely *equal* in the amount of attention bestowed upon them. The work requires especial commendation for the modest, unostentatious tone in which it is written; and the entire freedom from that assumption of originality, and oracular arrogance, which belongs to many who are not by any means as truly informed as he, as to what has been done every where by others. There is no attempt to place prominently forward any favorite school or *clique*, or to extol inordinately or invidiously what has been done by French rather than German—by German rather than English, and by all rather than by American observers, as we too often notice even on this side of the Atlantic. To Cæsar every thing that is due to him is rendered; and the reader cannot rise from the perusal of the work without being satisfied, that the author is an educated, well informed and liberal gentleman; and that his work is deserving of that place in the library of the student and practitioner for which it was originally destined by both author and publisher.

